# Toward AI foundation models for epidemics: Promise, challenges, and paths forward

**DOI:** 10.1073/pnas.2526192123

**Published:** 2026-03-13

**Authors:** Max S. Y. Lau, C. Jessica E. Metcalf, Zewen Liu, Bryan T. Grenfell, Wei Jin

**Affiliations:** ^a^Department of Biostatistics and Bioinformatics, Emory University, Atlanta, GA 30322; ^b^Department of Ecology and Evolutionary Biology, Princeton University, Princeton, NJ 08544; ^c^Department of Computer Science, Emory University, Atlanta, GA 30322

**Keywords:** epidemic foundation models, AI, machine learning, infectious disease

## Abstract

Foundation models—large AI systems pretrained on broad, heterogeneous data—are transforming scientific discovery. These models (e.g., GPT, GenCast, AlphaFold) excel at learning generalizable representations and adapting to new tasks with limited data. Yet, epidemic modeling has not experienced a comparable transformation. Traditional models remain pathogen-specific and often struggle to generate rapid insights during emerging outbreaks, as starkly illustrated by the SARS-CoV-2 pandemic. This Perspective asks whether the foundation model paradigm can extend to epidemic science: *Can we build a single, pretrained model that captures the shared principles of infectious disease dynamics across pathogens, populations, and settings?* Such a model could be fine-tuned to new contexts with minimal data, enabling faster forecasting, inference, and response, especially valuable in resource-limited settings. We argue that the growing convergence of epidemiological insight and modern AI makes this goal both urgent and increasingly plausible. We outline the main challenges in building foundation models for epidemics—nonstationarity, fragmented surveillance data, presence of diverse dynamical regimes, and the need for interpretability. We then propose a roadmap toward epidemic foundation models, emphasizing both algorithmic innovations to address these challenges and progress beyond algorithms, including investments in open datasets and cross-disciplinary training and collaboration. Developing epidemic foundation models offers a potentially transformative opportunity to strengthen global health security, particularly by improving preparedness in underresourced settings. If successful, they will serve as powerful, generalizable tools that complement existing efforts. The process of building these models will itself be valuable, exposing critical data gaps and guiding investments in global surveillance.

**Foundation models** (1)—large AI systems typically pretrained on broad, heterogeneous data—are transforming scientific research and biomedical practice. At their core, foundation models shift the emphasis from task-specific training to large-scale pretraining followed by efficient adaptation (or, fine-tuning) across a wide range of scientific and clinical tasks. First developed in domains like natural language processing and computer vision (2–4), foundation models now power breakthroughs in weather forecasting (5), geospatial modeling (6), financial forecasting (7), analysis of physiological biosignals (8, 9), and radiological diagnostics (10). Table 1 highlights representative foundation models in different domains with their primary capabilities. These models, which often depend on the availability of well-curated training corpora, excel at extracting generalizable patterns from broad, heterogeneous datasets and can deliver robust performance even in settings with limited labeled data. Box 1 offers a brief conceptual primer explaining key computational and epidemiological terms used throughout this Perspective.

**Table 1.** Representative foundation models in different domains

Model	Domain	Primary Capabilities
**GPT-4** (4)	Language	General-purpose language modeling across diverse domains.
**GenCast** (5)	Weather Forecasts	Weather forecasting, probabilistic prediction, shared atmospheric structure learning across space and time.
**AlphaEarth** (6)	Geospatial Modeling	Multi-task geospatial model that captures shared Earth system structure across space, time, and modalities.
**FinCast** (7)	Finance	Financial forecasting across diverse domains (stocks, crypto, forex) and resolutions; zero-shot generalization; probabilistic uncertainty modeling.
**PaPaGei** (8)	Photoplethysmography (PPG)	Estimation of cardiovascular health, sleep disorders, pregnancy monitoring, and well-being.
**ECG-FM** (9)	Electrocardiography (ECG)	Multi-label ECG interpretation, reduced LVEF screening, automated report benchmarking.
**BioGPT** (11)	Biomedical Language	Biomedical text generation, question answering, biomedical relation extraction.
**Galactica** (12)	Scientific Text	Molecule property prediction, chemical reaction prediction, citation prediction, etc.
**GatorTron** (13)	Clinical Language	Clinical concept extraction, classification, question answering, summarization.

Box 1. Conceptual Primer.

**Foundation Models**. Large AI systems typically pretrained on broad, heterogeneous datasets to learn broadly transferable representations. A core property of a foundation model is multitask generalization: after pre-training, the same model can be fine-tuned for tasks such as forecasting, inference, counterfactual reasoning, or simulation, typically requiring only limited task-specific data. In this Perspective, we use “epidemic foundation model” to refer to broad, cross-context generalization in complex dynamical epidemic systems, rather than text-focused adaptations of large language models.

**Pre-training and Fine-Tuning.** Pre-training refers to training a large model on diverse datasets to learn general and broad statistical and structural regularities in data. Fine-tuning then adapts these learned representations to a specific setting or task using limited additional data, enabling rapid and effective deployment in data-scarce settings.

**Few-Shot and Zero-Shot Performance**. Few-shot performance refers to a model’s ability to adapt to a new task using only a handful of labeled examples, while zero-shot performance denotes generalization to entirely new tasks without any additional training data. Strong few- or zero-shot performance is a key benchmark for evaluating epidemic foundation models, reflecting their ability to generate useful predictions for newly emerging pathogens with limited early information.

**Mechanistic Epidemic Models**. Cornerstone frameworks in infectious disease modeling that encode biological and behavioral assumptions about transmission. Classic examples include compartmental SIR or SIRS models, which describe transitions between susceptible (S), infected (I), and recovered (R) populations through mechanistic equations governing infection and recovery dynamics.

**Reproduction Number (R)**. A central measure of transmission potential: the expected number of secondary infections generated by a typical case.

Generalization has likewise been a central ambition of epidemic modeling. While existing epidemic models, ranging from compartmental frameworks (14, 15) to deep learning forecasters (16, 17), have proven useful, most are trained independently for a single pathogen in a specific setting. These models often require extensive tuning and struggle to generalize beyond their training context. The SARS-CoV-2 pandemic exposed these limitations starkly. Even with unprecedented global attention and data sharing, the modeling community often struggled to rapidly and accurately predict the ongoing spread of the novel pathogen, assess its severity and outcomes, and allocate medical resources effectively to minimize strain on healthcare systems, especially in the early stages of the pandemic (18, 19). Other recent examples include the re-emergence of measles in the United States (20) and the 2022 global spread of mpox (21), both of which highlighted the difficulty of building reliable models in settings where high-quality data are limited, incomplete, or rapidly evolving. Similar challenges arise more broadly in low- and middle-income countries, where surveillance systems are often sparse, inconsistent, or underresourced (22).

These gaps raise a pressing and timely question: *Can we build foundation models for epidemics, analogous to those that have transformed other fields* (Table 1)*, that are capable of learning from diverse outbreaks to enhance our understanding of transmission dynamics, improve forecasting and inform real-time public health responses, both for ongoing epidemics and newly emerging threats?* If feasible, such epidemic foundation models could offer a powerful way forward by complementing existing epidemic models, particularly through enabling generalization and effective deployment in resource-limited settings or rapidly evolving scenarios.

## The Vision: What Would an Epidemic Foundation Model Look Like?

**Fig. 1.**
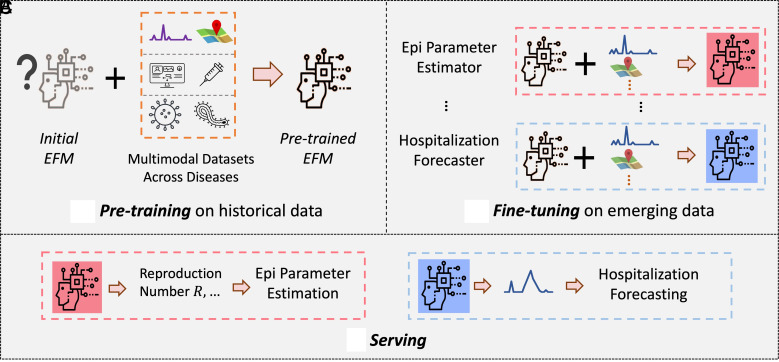
A blueprint of an epidemic foundation model (EFM). It operates in three stages: (*A*) pretraining on historical epidemic data across diverse diseases, locations, sources, and modalities; (*B*) fine-tuning on emerging data for specific settings and tasks, and (*C*) applying to target tasks for particular diseases.

At its core, an epidemic foundation model would learn from a diverse corpus of historical outbreaks to extract shared representations of transmission dynamics that ***generalize across pathogens, geographies, and populations***. As illustrated in Fig. 1, rather than training bespoke models for each disease or location, the aspiration is to pretrain a single transferable model on heterogeneous historical data. This pretrained model ***could then be fine-tuned with minimal, context-specific information***, a capability particularly vital during emerging outbreaks when reliable data are often scarce and time-sensitive.

The model would function as a ***data-fusion engine***, integrating heterogeneous and multimodal data streams to construct a coherent view of disease dynamics. These inputs could include, but are not limited to, time series of reported cases, pathogen genomic sequences, serological and wastewater surveillance, mobility and behavioral indicators, electronic health records, and records of public health interventions. Integrating these complementary sources would enable a unified system capable of capturing underlying mechanisms of spread across space, time, and biological scales.

Furthermore, a true foundation model for epidemics would move beyond forecasting case counts to serve as a comprehensive epidemiological toolkit. It would ***support a broader set of tasks*** such as estimating epidemiological quantities (e.g., effective reproduction number, undocumented infections); reconstructing transmission networks; simulating counterfactual scenarios under hypothetical interventions; forecasting evolutionary trajectories; and producing uncertainty-calibrated projections.

Rather than replacing classical epidemic models such as the SIR family, which already provide a high-level conceptual framework capturing some shared mechanistic insights across pathogens, an epidemic foundation model would seek to *extend this pursuit of generality into the data-driven domain*. Pretraining enables the model to learn shared structure across diverse pathogens, transmission settings, and data modalities, while fine-tuning provides pathogen- or context-specific adaptation—analogous to the traditional practice of calibrating mechanistic models for particular diseases. In this sense, *epidemic foundation models* ***offer a broader form of generalization***: They assimilate knowledge across heterogeneous real-world scenarios to establish a shared baseline representation, from which models can learn, adapt, and respond to new pathogens or settings by leveraging prelearned dynamical patterns, potentially reducing the search space for downstream adaptation to ongoing spread compared to training from scratch. Conceptually, epidemic foundation models, through pretraining and fine-tuning, might offer a pathway to balance and integrate the strengths of both (“simple”) generalizable and (“complex”) precise/tactical models, a trade-off long recognized in epidemiology and ecology (23, 24). Ultimately, if realized, epidemic foundation models could provide more than a technical advance: They may help shape a new organizing principle for epidemic science, in which shared knowledge from past outbreaks strengthens preparedness and agility for future epidemics.

## Challenges for Developing Epidemic Foundation Models

**Fig. 2.**
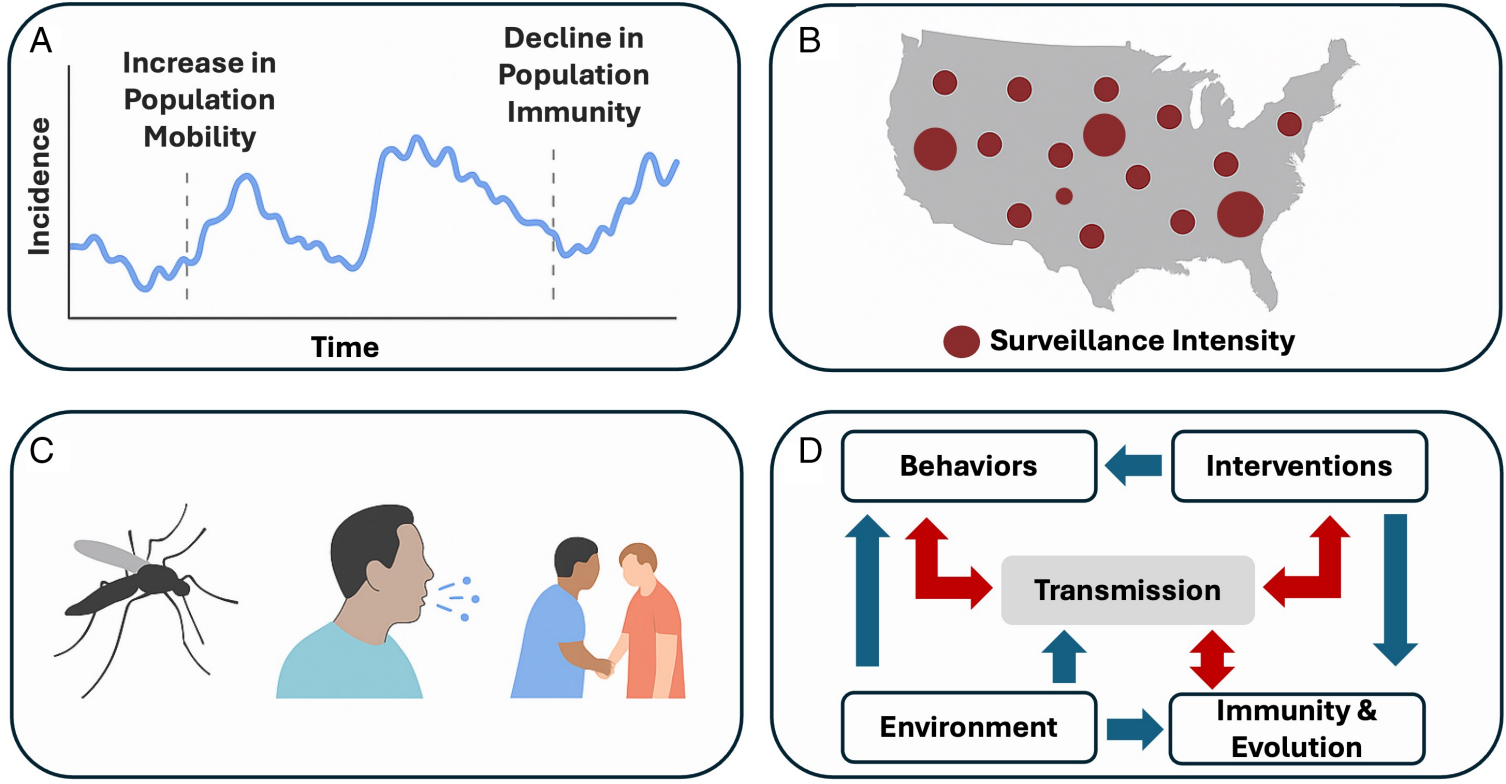
Challenges of developing epidemic foundation models. (*A*) Nonstationary dynamics due to factors such as changes in population movement and immunity. (*B*) Data sparsity and fragmentation, for example, due to uneven surveillance intensity and inconsistent reporting across regions. (*C*) Presence of diverse dynamical regimes due to, for example, differences in modes of transmission between pathogens. (*D*) Complex causal or mechanistic relationship, including potential feedback loops (red arrows) among shifts in epidemiological, behavioral, immunological, evolutionary, and environmental factors.

Epidemic systems differ in fundamental ways from many domains where foundation models have flourished (Fig. 2). A first key distinguishing feature is the ***nonstationarity*** of epidemic dynamics. Unlike domains such as natural language, which tend to follow relatively stable distributional patterns, epidemics are driven by processes whose statistical properties evolve over time. A close scientific analog is weather forecasting, where models such as GenCast (5) must accommodate shifting atmospheric regimes and evolving environmental conditions. Yet, the sources of nonstationarity in epidemics are arguably even more complex (and often more rapid), arising not only from environmental or seasonal drivers but also from more immediate feedbacks between biological, behavioral, and policy processes. For instance, human behavior changes with risk perception or policy (25), population immunity evolves through infection, vaccination, or waning protection (26), and public health actions can abruptly alter contact structure and reporting practices (27).

This continual interaction creates a highly dynamic data-generating process. As a result, even models that perform well on historical data may produce confidently inaccurate predictions when applied to new settings or future periods. In this sense, epidemic modeling must contend not only with nonstationary data but with nonstationary processes, where the underlying generative mechanisms themselves can shift. Addressing this challenge requires models that can detect changing regimes and adapt as epidemiological conditions evolve.

Foundation models are typically pretrained on large, dense, and well-curated datasets. Although the global volume of data is increasing, epidemic data at the scale of individual outbreaks continue to face distinctive challenges of ***sparsity and fragmentation***. Specifically, epidemiological data are often incomplete, inconsistently reported, highly variable across settings and lack standardized metadata (28–31). For example, temporal coverage or reporting rate is uneven, and surveillance quality differs markedly across countries and regions. Metadata, such as geographic identifiers used in routine epidemiological surveillance, often lack standardization (e.g., two-letter vs. three-letter country codes, state name variations). Temporal formats also vary—for example, some use specific dates while others report by week number. In contrast to fields like computer vision and natural language processing, where the development of foundation models has been propelled by curated corpora, epidemic modeling lacks standardized datasets of comparable scope. This absence hinders effective model pretraining and reproducible evaluation, making it difficult to develop, benchmark, or compare models systematically.

Another central challenge is the ***presence of diverse dynamical regimes***, shared with other domains governed by physical laws (e.g., weather), that arise across pathogens. Diseases such as dengue, COVID-19, and measles exhibit markedly different epidemiological and evolutionary dynamics (32–36). These contrasts arise from both biological heterogeneity, including mutation rates, immune escape potential, and host susceptibility, and epidemiological contexts such as mode of transmission. For instance, dengue is governed by vector-borne, multiserotype dynamics, where host-vector coupling, climatic forcing, and immune-mediated feedbacks jointly shape transmission (37); SARS-CoV-2 is characterized by rapid antigenic evolution under immune pressure (38); and measles exhibits low-dimensional chaotic dynamics arising from nonlinear susceptibility depletion in partially immune populations (39). A foundation model for epidemics must therefore learn generalizable representations that capture shared transmission principles (e.g., susceptibility depletion as predicted by the SIR model family), while also accommodating qualitatively and structurally different dynamical regimes arising from heterogeneous and intrinsic mechanisms that govern different pathogens.

The challenge of generalization is further compounded by ***causal and mechanistic interdependence*** among multiple drivers. This challenge is shared by many scientific domains, in which identifying causal structure in large, interconnected systems remains a major open problem due to confounding, indirect causation, and limited observability. Epidemic dynamics are highly nonlinear, shaped by complex interactions—and often feedback loops—among transmission processes, evolutionary dynamics, environmental conditions, behavioral responses to outbreaks, and public health interventions (40, 41). For example, a decline in reported case counts may reflect a true reduction in transmission, seasonal effects, changes in testing behavior, or a combination of these factors. These interactions also operate across scales—for instance, within-host viral evolution can alter the probability of transmission between hosts (42). Disentangling such influences may require models that can reveal underlying causal structure—not merely statistical associations. Relatedly, the ***imperative for interpretability*** is therefore more acute in epidemic modeling than in many other domains. Deep learning models excel at extracting statistical patterns and can yield accurate forecasts, but often at the cost of interpretability and transparency—an acceptable tradeoff in settings where predictive performance alone suffices, but not in public health, where decisions must be grounded in causal understanding (e.g., whether rising incidence reflects waning immunity, behavioral change, or the emergence of a new variant, each of which demands a different intervention). In contrast, traditional epidemiological models, such as compartmental frameworks (14), are built around mechanistic assumptions (e.g., contact rates, waning immunity) that are explicitly interpretable and directly actionable for policy. Epidemic foundation models must therefore navigate a “*tension”* between the appeal of purely scale-driven, general-purpose pattern learners [in the spirit of Sutton’s “Bitter Lesson” (43)] and the domain’s need for mechanistic insights to support interventions and counterfactual reasoning. They must learn flexible, data-driven representations that support accurate forecasting while retaining the capacity to expose mechanistic structure. This ***mismatch between purely black-box architectures and mechanistic traditions*** underscores the need for hybrid, semimechanistic approaches that combine the representational/predictive power of modern AI with the domain relevance and causal transparency required for real-world decision-making.

## Why Is an Epidemic Foundation Model Becoming Possible?

Despite these significant challenges, the vision for an epidemic foundation model is anchored in core epidemiological principles and supported by rapidly advancing computational capabilities, making it not merely aspirational but increasingly feasible.

***First***, while diseases differ in etiology, transmission mode, and evolutionary dynamics, they also share common drivers of spread, such as seasonality, population mobility, contact structure, and interventions like vaccination and social distancing. Across these diverse systems, a degree of dynamic, often oscillatory, regularity (or, “simplicity”) constantly emerges at the population level: Most infections broadly follow a susceptible–infected–recovered–susceptible (SIRS) progression mechanism (15), governed by a finite set of parameters such as transmission rate, immunity duration, and contact intensity. These recurrent features provide the scaffolding on which generalizable representations of epidemic dynamics can be learned.

In this respect, epidemic modeling parallels other domains where foundational structure enables pretraining to extract broadly transferable representations. Advances in representation learning, multimodal data integration, and scalable computing now make it possible to harness this shared epidemiological structure across pathogens and contexts. The development of epidemic foundation models, therefore, rests on a fundamental premise: *Despite biological, behavioral, and clinical heterogeneity, infectious disease dynamics exhibit consistent, learnable regularities (simplicities) grounded in well-established epidemiological principles.*

***Second***, although individual outbreak datasets remain sparse and fragmented, the global epidemiological data landscape has expanded dramatically. Broad, heterogeneous datasets, spanning historical outbreak records, genomic surveillance, serology, mobility, wastewater, and environmental covariates (29, 30, 44–47), are now available across many pathogens and regions. While none of these data streams are sufficient on their own, the cumulative breadth of these imperfect datasets makes epidemic foundation models increasingly more plausible than even a decade ago. This breadth provides a foundation on which generalizable representations can begin to be learned, even if substantial gaps remain. Equally important, the *growing adoption of open data standards* is beginning to make these resources interoperable, laying the groundwork for scalable pretraining and reproducible evaluation across pathogens and regions. At the same time, the persistent sparsity and fragmentation of individual datasets (see “*Addressing data sparsity and fragmentation*”) underscore the need for methods capable of learning from noisy, incomplete, or weakly labeled data.

**Fig. 3.**
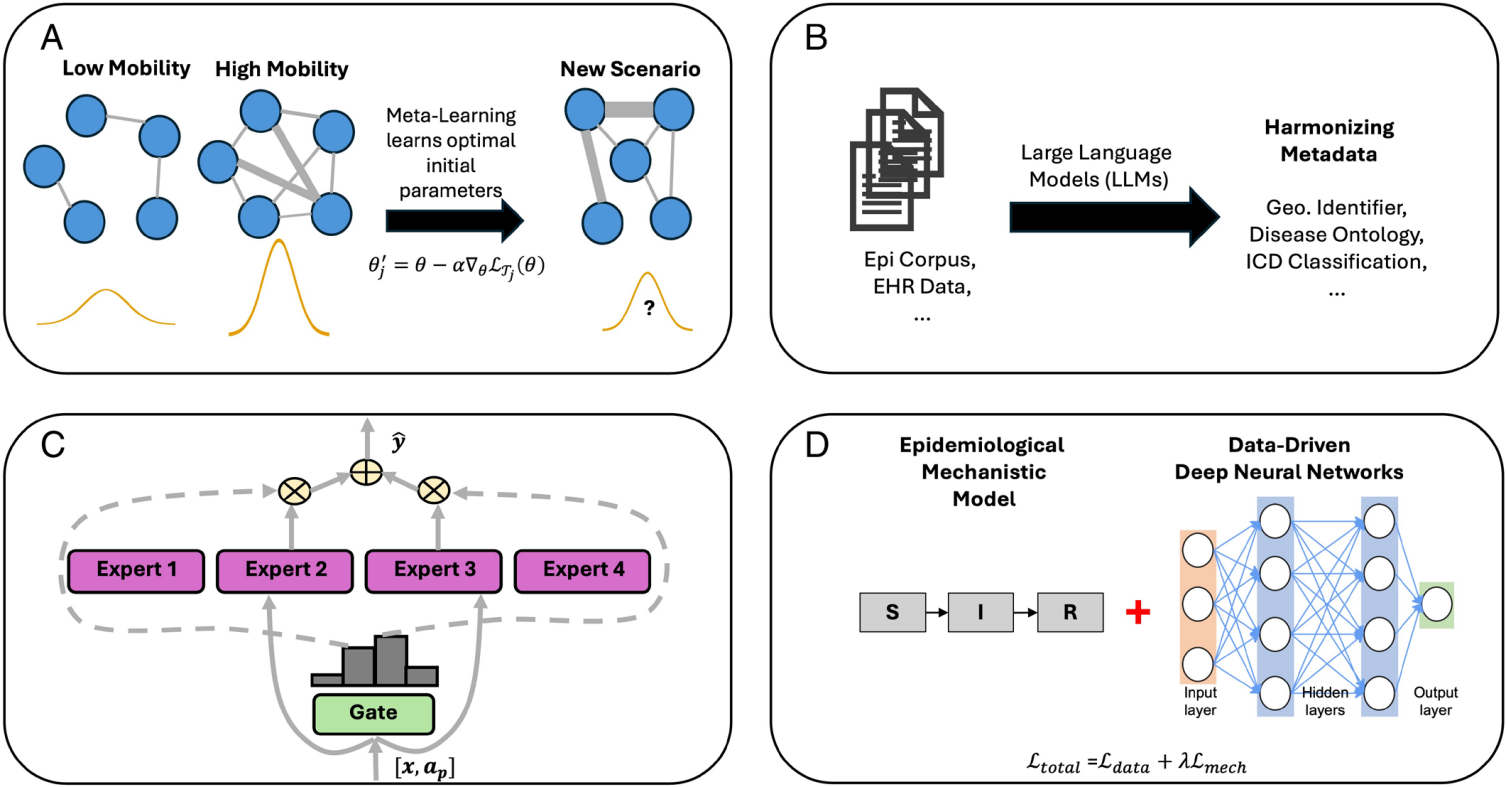
Examples of key components underpinning epidemic foundation models. (*A*) Meta-learning learns from a diverse set of outbreak scenarios, accommodating potentially rapidly changing and nonstationary transmission dynamics. Nodes represent locations, and edges represent mobility-informed connectivity. (*B*) Large language models (LLMs) harmonize inconsistent metadata across multiple sources of disease data. (*C*) An “expert” in a Mixture of Experts (MoE) framework specializes in a specific class of pathogen transmission regimes, with a “gate” network that shares, selects, or combines the outputs from the experts. (*D*) Epidemiologically aware hybrid frameworks combine traditional S-I-R models with data-driven deep learning models, ensuring that the overall framework is epidemiologically grounded and mechanistically interpretable.

***Third***, *recent works* (48, 49) *have demonstrated both the proof-of-concept and early empirical evidence for the feasibility and promise of building an epidemic foundation model.* For instance, CAPE (48), developed by the authors, introduced one of the first pretrained epidemic models leveraging transformer architectures (2, 50). CAPE was pretrained on 17 distinct diseases (12 respiratory and 5 nonrespiratory) and fine-tuned on five unique diseases spanning over diverse regions, which are collected from multiple global surveillance sources. It demonstrated that a single pretrained backbone could be fine-tuned to forecast infections for multiple pathogens, including emerging ones such as COVID-19 that were unseen during pretraining. More specifically, CAPE learns a flexible dictionary of latent compartmental prototypes and employs epidemic-aware self-supervised losses to align learned disease dynamics with epidemiological principles. Similarly, PEMs (49) share a related motivation and framework, focusing likewise on infection forecasting through large-scale pretraining to capture transferable epidemic dynamics. While these works highlight the feasibility of epidemic-specific foundation models and underscore the need for continued innovation in this space, they remain focused on a single epidemic task (i.e., infection forecasting) and still face key challenges of developing a robust epidemic foundation model, including nonstationary dynamics, data sparsity, presence of diverse dynamical regimes, and interpretability (outlined in Fig. 2). In the next section, we discuss several directions (Fig. 3) for overcoming these challenges to advance the next generation of epidemic foundation models.

## Promising Directions for Building Epidemic Foundation Models

### Addressing Nonstationary Dynamics.

To counter nonstationarity, foundation models must *incorporate adaptive learning mechanisms* that allow them to adjust as epidemic conditions evolve. One promising approach is metalearning (51, 52), which explicitly trains models to quickly adapt to new outbreak conditions from limited observational data. In this framework, the model is trained not on a single epidemic but on a diverse set of simulated or historical outbreak scenarios $\left\{T_{1},T_{2},\cdots ,T_{N}\right\}$, which vary in transmissibility, seasonality, intervention timing, population structure, and population mobility. A concrete illustration is mobility-driven nonstationarity represented on a dynamic contact or transportation network: Each scenario can include time-varying adjacency matrices A(t) whose edge weights track travel flows or mixing intensity. As mobility patterns shift, the resulting changes in network connectivity induce changing transmission regimes T that the model must learn to accommodate. For each scenario $T_{j}$, the model first adapts its parameters θ to a small support set of early observations, producing updated parameters $\theta_{j}^{\prime }=\theta -\alpha \nabla_{\theta }L_{T_{j}}\left(\theta \right)$, where α controls the adaptation speed and $L_{T_{j}}$ measures forecast error. The metalearning objective then optimizes the original parameters θ so that, after this quick update, the adapted model performs well across all outbreak scenarios: $\min_{\theta }\sum_{j}L_{T_{j}}\left(\theta_{j}^{\prime }\right)$. In general, through exposure to scenarios with dynamic networks, seasonal forcing, and shifts in human behavior, the model learns a general strategy for rapid few-shot adaptation—enabling it to recognize and adjust to these classes of nonstationarity using only limited new data.

A complementary way to handle nonstationarity is to directly evolve model parameters over time, allowing them to capture gradual changes in transmission intensity, behavior, or environment. Formally, this can be expressed as a dynamic update: $\theta_{t+1}=h\left(\theta_{t},x_{t},\eta_{t}\right)$, where h(·) governs how parameters evolve given new data $x_{t}$ and contextual factors $\eta_{t}$. Here, $\eta_{t}$ can explicitly encode mobility features such as network-level centrality shifts, seasonal covariates such as humidity or temperature, and indicators of adaptive human behavior such as adherence to distancing or masking policies. Model updating strategies such as continual learning (53, 54) could allow the model to recalibrate its understanding as fundamental epidemiological drivers change. This paradigm enables the model to incrementally learn from a continuous stream of new data, capturing events like a new variant’s emergence or a policy change, while actively preserving core knowledge from past periods.

### Addressing Data Sparsity and Fragmentation.

Addressing this challenge begins with improving the quality and consistency of the available observations. *Advanced augmentation and imputation methods* (55–57) can infer missing values within existing datasets by leveraging learned patterns and correlations, while computational pipelines for curating and standardizing metadata (58, 59) ensure that information from heterogeneous sources can be merged reliably. Recent progress in *large language models* (*LLMs*) (4, 60–62) provide a powerful complement: Trained on massive corpora of biomedical and epidemiological text, they can infer plausible missing information from partial or noisy records, or synthesize structured representations from unstructured sources such as electronic health records (EHRs), case reports, and surveillance bulletins. When integrated into preprocessing pipelines, these tools can help fill gaps in patient histories, align mismatched metadata fields, and produce more coherent datasets that strengthen the downstream learning signal for epidemic foundation models.

Once data are reconstructed and standardized, *transformer-based architectures* (2, 50, 63) provide a natural modeling backbone, since their attention mechanisms can fuse irregular time series, spatial information, and multimodal inputs while retaining robustness to missing entries and multiscale structure. To fully exploit partially observed data, designing more *effective self-supervised* (64, 65) *or semisupervised paradigms* (66–68) becomes crucial. For instance, a self-supervised approach could involve training a transformer on a pretext task, such as predicting intentionally masked data points within an epidemic time series, which forces it to learn the underlying temporal dynamics without any external labels. Furthermore, consistent with the strategies proposed for handling nonstationary dynamics (see previous subsection), *metalearning and few-shot learning approaches* (51, 52) offer a tangible path for rapid adaptation. By treating different historical outbreaks as distinct scenarios, a metalearning framework allows the model to learn initial parameters that can be quickly fine-tuned to a new, data-sparse epidemic context, i.e., via few-shot adaptation. In parallel, semisupervised pipelines can leverage a small set of high-quality labeled data (e.g., from well-resourced regions) to generate pseudolabels for large collections of unlabeled data, thereby expanding the usable training dataset to improve generalization across data-scarce environments.

Beyond improving existing data, *large-scale synthetic data simulations*—particularly those built on the rich body of models grounded in epidemiological principles and calibrated with real outbreak data (69–71)—can play a critical role in augmenting sparse real-world observations. These simulations not only expand the diversity of epidemic scenarios available for model training but also enable systematic experimentation under controlled conditions, where causal mechanisms and parameters are known. Synthetic epidemic corpora can incorporate a wide range of generative models—from agent-based simulations that capture fine-grained contact structures and behavioral feedbacks, to stochastic compartmental or metapopulation frameworks that reproduce large-scale transmission dynamics. By varying parameters such as reproduction number, seasonality, spatial coupling, or intervention timing, these simulated datasets can span the full spectrum of outbreak regimes, providing a rich substrate for model training and evaluation.

Similar paradigms have already proven transformative across several scientific domains–for example, in molecular biology, physics-based simulations of protein folding generate massive synthetic corpora that underpin models like AlphaFold (72). For epidemic foundation models, such simulations can provide pretraining data when labeled observations are scarce, stress-test robustness under novel conditions, and generate counterfactual scenarios that are impossible or unethical to observe empirically. When designed to mirror real surveillance structures, they can produce synthetic case counts, genomic sequences, or mobility patterns in consistent formats, allowing seamless integration with real datasets throughout pretraining, fine-tuning, and evaluation. In doing so, synthetic data serve as both a bridge and a scaffold for developing generalizable epidemic foundation models.

### Addressing Diverse Dynamical Regimes.

Potential solutions involve developing modular foundation models capable of integrating pathogen-specific dynamics or learning adaptive parameters unique to each infectious agent or its variants. *Mixture of Experts* (MoE) (73, 74) models offer a promising architectural approach: They consist of multiple expert subnetworks, each specializing in different classes of transmission dynamics, along with a gate network that learns to select, route, or combine the outputs of the most relevant experts for a given input. In mathematical form, the model’s prediction can be written simply as $\hat{y}=\sum_{i}g_{i}f_{i}(x)$, where $f_{i}\left(x\right)$ is the output of expert i and $g_{i}$ is the gate’s weight indicating its relevance for the current input x. This structure allows the model to “switch on” the most relevant experts for a given pathogen while still learning common patterns across all of them. This enables the foundation model to dynamically activate expert pathways suited to specific pathogen types, while capturing shared patterns and dynamics within a unified framework. As a complementary strategy, the gating and expert networks can be guided by structured pathogen descriptors such as mode of transmission and typical incubation period, or basic reproduction number. Representing these as a compact feature vector $a_{p}$ for pathogen p, we can condition the experts and gates on both x and $a_{p}$, e.g., $f_{i}\left(x,a_{p}\right)$ and $g_{i}\left(x,a_{p}\right)$. This provides the model with strong, biologically grounded context, thus improving generalization to new or evolving pathogens.

Importantly, such MoE architectures have already demonstrated remarkable scalability and specialization in other scientific and engineering domains such as large language models (62, 75, 76), protein representation learning (77, 78), and medical analysis (79), where they enable systems to manage vast heterogeneity without sacrificing efficiency. Their success suggests similar potential for epidemic modeling, where distinct pathogens or transmission modes may benefit from expert specialization within a unified model.

### Addressing Complex Causal Relationships and Ensuring Interpretability.

This requires foundation models capable of inferring and representing causality beyond mere correlation. *Natural experiments in disease epidemiology and ecology provide valuable opportunities to test and refine causal reasoning in an epidemic foundation model*. For example, the seasonal timing of school terms has long served as a quasiexperimental setting for understanding measles transmission dynamics and the role of contact structure (80). Similarly, the significant reduction or increase in birth rates during certain periods has helped disentangle the role of demographic turnover in epidemic cycles. Vaccination campaigns also provide a mechanistic lens for understanding how structured interventions perturb stationary transmission dynamics, exposing key drivers of transmission dynamics (81).

To systematically operationalize these insights, two emerging approaches are particularly relevant. First, *causal discovery under latent confounders* (82, 83) can be employed to infer novel directional relationships and causal structures directly from sparse observational data. Second, to explicitly incorporate established epidemiological principles, hybrid mechanistic-data-driven frameworks offer a robust path forward. Recent development in *epidemiology-aware deep neural networks* (84–86), for example, enables a mechanism for integrating the structured knowledge from the natural experiments, thus providing crucial causal constraints and mechanistic interpretability. These models integrate mechanistic assumptions directly into the neural network architecture or its training objective. The standard data-driven loss $L_{\mathrm{data}}$ is augmented with a mechanistic constraint loss ($L_{\mathrm{mech}}$), guiding the model’s internal representations: $L_{\mathrm{total}}=L_{\mathrm{data}}+\lambda L_{\mathrm{mech}}$. Here, $L_{\mathrm{mech}}$ penalizes the epidemic foundation model if its internal latent rates or states violate established epidemiological laws, such as the nonnegativity of inferred transmission rates (β≥0) or susceptible depletion. Incorporating such epidemiology-aware deep neural networks allows the foundation model to learn relationships that align with established biological and epidemiological understanding, thereby guiding the learning process for epidemic foundation models toward causally sound representations.

Furthermore, to make the EFM’s insights more interpretable and actionable, we must *integrate robust Explainable AI (XAI) methods* such as Shapley values (87, 88). These methods provide quantitative attribution scores, revealing the precise contribution of every input feature (e.g., a 15% increase in mobility, the timing of a vaccination campaign, or a change in climate) to a specific forecast or inferred quantity (e.g., next week’s reproduction number). This transparency is essential for informing high-stakes policy decisions, allowing decision-makers to understand why a prediction was made, which builds the trust necessary for field deployment.

### What Is Needed beyond Algorithms?

Unlocking the potential of foundation models for epidemics will require more than advances in algorithms. Progress at scale depends on key infrastructural and community-level investments to ensure that models are scientifically credible, broadly applicable, and transparent.

### Data Standardization and Access.

There is an urgent need to establish standardized, open-access epidemic datasets that span diseases, geographies, and time. While initiatives such as Project Tycho (44) have made substantial progress in harmonizing historical surveillance data, epidemiological data remain largely dispersed across multiple sources, inconsistently formatted, and poorly annotated, making large-scale integration difficult (28–30). Scaling existing initiatives across pathogens, geographies, and data modalities, analogous to shared corpora in language (2) or vision (89), will be critical for training and benchmarking epidemic foundation models (90).

Public data repositories should integrate multiple data modalities relevant to transmission dynamics, including reported cases, hospitalizations, and deaths; genomic and serological data; wastewater data; environmental and climate indicators; and records of public health interventions such as vaccination and school closures. Adopting open metadata standards following FAIR principles (91) (Findable, Accessible, Interoperable, Reusable) would promote interoperability and reproducibility across research groups. Each dataset must include clear metadata describing spatial and temporal coverage, data provenance, consistent geographic identifiers, harmonized intervention annotations, and, where applicable, linkages between datasets through shared sample or case identifiers to facilitate integration across epidemiological, genomic, and environmental data streams (34, 92). Public repositories should also provide version-controlled, API-based access with transparent update logs to ensure traceability and long-term usability.

While some relevant data, such as clinical records, mobility traces, and detailed contact patterns, are sensitive or privately held, their inclusion can also accelerate the development of comprehensive epidemic foundation models. Progress will depend on developing privacy-preserving data-sharing frameworks [e.g., federated learning (93), differential privacy (94), design of secure data sharing environments (95)] that enable model training across decentralized datasets without compromising confidentiality. Establishing standardized access protocols and governance agreements between data providers, health agencies, and researchers will be critical.

### Shared Benchmarks and Milestones.

In parallel, the field must establish shared benchmarks and evaluation protocols. Without consensus standards for assessing model performance, across tasks such as forecasting, inference, and simulation, progress will remain fragmented and difficult to measure.

Benchmarking initiatives should emphasize not only predictive accuracy but also model reliability under distribution shift, which is inevitable in epidemic systems due to nonstationary drivers such as behavioral adaptation, variant emergence, or policy changes. Evaluating model performance solely on in-sample forecasts risks overestimating robustness; benchmarks must therefore include deliberate stress tests that assess how rapidly and safely a model can adapt when the underlying transmission process changes. This could include, for example, training on prevariant COVID-19 data and evaluating on a period dominated by the Delta or Omicron variants or simulating abrupt changes in reporting or intervention intensity to probe stability.

Beyond simple predictive metrics, benchmark frameworks should include quantitative measures of uncertainty calibration and interpretability. Well-calibrated uncertainty, assessed using proper scoring rules such as WIS (Weighted Interval Score) (69, 96), ensures that forecasted CI meaningfully reflect real-world variability. Complementary interpretability benchmarks should test whether a model’s attributions align with established epidemiological understanding—for instance, whether inferred contact-rate fluctuations correspond to actual mobility shifts or intervention changes.

Equally critical is assessing a model’s capacity for generalization: i) few-shot and zero-shot adaptation to novel outbreaks with minimal new data; ii) cross-pathogen transfer, where a model pretrained on one class of respiratory infections is evaluated on a distinct pathogen family; and iii) geographic transfer, evaluating skill retention when deployed in regions with sparse surveillance. Success in these settings would demonstrate that the model has captured the shared structural features of epidemic dynamics, rather than pathogen- or location-specific patterns.

Benchmarks should also include counterfactual validity—the ability to simulate unseen interventions or behavioral scenarios and yield epidemiologically plausible outcomes. For example, shifting a vaccination campaign forward by 4 wk or introducing school closures should produce coherent and quantitatively reasonable changes in predicted epidemic trajectories. Similarly, evaluating whether models can recover known mechanistic signatures [e.g., birth rate–driven measles cycles (80), predictable biennial RSV oscillations (97)] provides a mechanistic validation that their learned representations respect real epidemiological constraints.

Collectively, these benchmark categories provide measurable milestones for progress toward epidemic foundation models, enabling the community to track advances in generalization, transfer, causal inference, and multimodal integration.

### Cross-Disciplinary Collaboration and Training.

Equally important is fostering cross-disciplinary collaboration. Building effective foundation models for epidemics requires integrating methodological advances from machine learning and AI with domain expertise in epidemiology, evolutionary biology, public health, computer science, and behavioral science. Yet, these communities often operate in silos, with distinct vocabularies, data practices, and publication norms. Overcoming these divides is essential to ensure that models are both technically robust and epidemiologically meaningful.

In practice, this means developing purpose-built collaborative structures. Interdisciplinary consortia can serve as neutral spaces where AI researchers and domain scientists codesign model architectures, evaluation metrics, and data standards. Similarly, embedded fellowship programs—for instance, placing data scientists within public health agencies or epidemiologists within computer science labs—can facilitate reciprocal learning and foster a shared understanding of practical constraints.

Training initiatives will also be critical. Graduate and postdoctoral curricula could incorporate “hybrid” courses covering mechanistic modeling, infectious disease ecology and epidemiology, and modern AI architectures, enabling the next generation of researchers to move fluently across domains. Workshops and summer courses [e.g., The Summer Institute in Statistics and Modeling in Infectious Diseases (SISMID) (98) or NeurIPS-style tutorials (99)] could provide intensive, hands-on exposure to cross-disciplinary methods and datasets.

Finally, joint funding mechanisms are needed to sustain these collaborations. Multidisciplinary calls from national and international agencies could explicitly support teams that combine technical and domain expertise, encouraging coleadership between computer scientists, modelers, and epidemiologists. Importantly, collaboration should extend beyond academia to include public health practitioners and policymakers, ensuring that foundation models are aligned with decision-making needs and ethical considerations.

Only through such sustained cross-disciplinary engagement can the field build epidemic foundation models that are scientifically rigorous, operationally relevant, and trusted by the communities that depend on them.

### Transparent Decision-Support Systems.

Finally, the modeling community and public health agencies must jointly develop transparent decision-support systems. For foundation models to effectively inform outbreak preparedness, vaccination policy, and resource allocation, their outputs must remain interpretable and uncertainty-aware.

Models should communicate uncertainty and assumptions clearly, not only through numerical intervals but through brief, epidemiologically grounded explanations of why forecasts change (e.g., shifts in testing, mobility, or intervention timing). Transparent communication helps prevent overconfidence and enables decision-makers to weigh model outputs alongside other evidence. Each model should include documentation detailing its intended scope, key assumptions, and limitations, together with its changelogs and data provenance.

Long-term sustainability will depend on consortium-based governance that brings together public health agencies, academic institutions, and international partners such as WHO. Such collaboration can ensure consistent evaluation, ethical oversight, and equitable access to modeling tools.

Ultimately, epidemic foundation models should augment rather than replace expert judgment, providing interpretable, uncertainty-calibrated insights that inform, but never dictate, public health decisions. Embedding transparency from design to deployment will be essential for maintaining public trust and realizing the promise of AI-driven decision support in epidemic response.

## Conclusion

The development of foundation models for epidemics holds great promise, but fulfilling that promise will require models that are deeply attuned to the unique demands of outbreak response and management. Epidemic foundation models must contend with nonstationary processes, sparse and fragmented data, presence of diverse dynamical regimes, and the imperative for deep interpretability. If successfully developed, these models could significantly strengthen epidemic preparedness by complementing existing modeling efforts, particularly in resource-constrained settings or during rapidly evolving outbreaks where timely, generalizable insights are critical for decision-making.

Even partial success of such foundation epidemic models with current data could yield important benefits by revealing the most consequential data gaps and guiding targeted investments in surveillance systems. This iterative process—using models to inform data collection, which in turn improves the models—would accelerate progress toward truly generalizable epidemic models transferable across pathogens. The same logic underpins other ambitious public health goals, such as the pursuit of a universal influenza vaccine (100), where understanding cross-strain immune responses has been essential to designing broadly protective strategies.

Progress will depend not only on algorithmic advances but also on investments in open datasets, benchmarking standards, and cross-disciplinary infrastructure. Early work such as CAPE demonstrates the feasibility of epidemic-specific pretraining, offering a blueprint for what is possible. Moving forward, success will depend on building models that are not only scalable and generalizable but also transparent, trustworthy, and actionable in real-world public health settings. Only then can foundation models serve as reliable tools for epidemic preparedness and response.

## Data Availability

There are no data underlying this work.
